# Antioxidant assays – consistent findings from FRAP and ORAC reveal a negative impact of organic cultivation on antioxidant potential in spinach but not watercress or rocket leaves

**DOI:** 10.1002/fsn3.71

**Published:** 2013-10-16

**Authors:** Adrienne C Payne, Alice Mazzer, Graham J J Clarkson, Gail Taylor

**Affiliations:** 1 Centre for Biological Sciences, University of SouthamptonLife Sciences Building, Highfield Campus, Southampton, Hampshire, SO17 1BJ, United Kingdom; 2 Vitacress Salads LtdLower Link Farm, St Mary Bourne, Andover, Hampshire, SP11 6DB, United Kingdom

**Keywords:** Brassicaceae, leafy salad crops, organic agriculture, rocket, spinach, watercress

## Abstract

Watercress (*Rorippa nasturtium-aquaticum*), wild rocket (*Diplotaxis tenuifolia*), and spinach (*Spinacia oleracea*) are commercial crops reported to have high concentrations of antioxidants, possibly contributing to disease prevention following human consumption. Following analysis of supermarket-purchased salad leaves, we report the antioxidant content potential of these species using two comparable techniques assessing the consistency between the assays – by the ferric reducing antioxidant power (FRAP) assay and the oxygen radical absorbance capacity (ORAC) assay. The leaves were harvested from both conventionally and organically managed crops, to investigate whether organic agriculture results in improved crop quality. Watercress had the highest FRAP and ability to scavenge free radicals, followed by spinach and rocket. For watercress and rocket, there was no significant effect of organic agriculture on FRAP and ORAC, but for spinach, the antioxidant potential was reduced and this was significant at the 5% level of probability for FRAP but not ORAC, although the trend was clear in both tests. We conclude that there is variation in salad crop antioxidant potential and that FRAP and ORAC are useful techniques for measuring antioxidants in these salad crops with similar ranking for each salad crop studied.

## Introduction

In recent years, interest in the antioxidant status of plant-derived foods has risen due to the potential health benefits from their ability to scavenge radicals (Podsedek 2007). A free radical is a free atom, molecule, or ion which contains one or more unpaired electrons (Aruoma et al. 1993). Free radicals play an important role in a number of biological processes but can react with and damage DNA, causing many forms of cancer (Traka and Mithen 2009). The term “antioxidant” covers a wide range of different molecules but a common feature is their ability to readily donate electrons while remaining stable themselves, hence acting as reducing agents (Aruoma 1998) and minimizing damage caused by free radicals.

Antioxidants present in plants (acting as a defense system within the plant) can deactivate radicals by two major mechanisms: hydrogen atom transfer (HAT) and single electron transfer (SET), although both produce a similar end result (Prior et al. 2005). Many antioxidant protocols have been devised to determine the antioxidant status of foods. Of particular interest are the ferric reducing ability of plasma/ferric reducing antioxidant power (FRAP) utilizing the SET reaction and oxygen radical absorbance capacity (ORAC) utilizing the HAT reaction (Prior et al. 2005), but their relationship with each other has rarely been investigated.

Watercress (*Rorippa nasturtium-aquaticum*), a member of the Brassicaceae family, contains a wide range of natural, bioactive plant compounds (phytochemicals) for which there is increasing evidence of beneficial effects to human health (Rose et al. 2005; Gill et al. 2007). Watercress contains one of the highest concentrations of glucosinolates per gram weight of any vegetable as well as carotenoids such as leutin and β-carotene (Gill et al. 2007). Glucosinolates are precursors of isothiocyanates and upon chewing watercress phenethyl isothiocyanate is released (Hecht et al. 1995). Gill et al. (2007) highlighted the benefits of watercress consumption as a reduction in lymphocyte DNA damage was observed, which could be linked to a reduced risk of cancer. Spinach (*Spinacia oleracea*) has been shown to have an exceptionally high phenolic content and high total antioxidant activity (Hecht et al. 1995; Gil et al. 1999; Ismail et al. 2004). Wild rocket (*Diplotaxis tenuifolia*) is a source of carotenoids (lutein, β-carotene), vitamin C, folate, flavonoids, glucosinolates (glucoerucin), and fiber (Barillari et al. 2005; Hedges and Lister 2005).

Here we focused on a simplified adapted ORAC protocol (Zullo and Ciafardini 2008) and an adapted FRAP assay (Benzie and Strain 1996), to consider the ability of the antioxidants present in the salad samples to scavenge radical species and to reduce Fe(III)/tripyridyltriazine complex. Both methods are reported to reflect total antioxidant activity and results from each should be comparable despite the different reaction mechanisms involved in each assay (Cao et al. 1995; Ou et al. 2002), although this has rarely been tested.

The aim of this study was first to compare the antioxidant potential of three salad crops (watercress, spinach, and wild rocket) widely grown in Europe. Second, we wished to understand if different assays gave similar findings for both magnitude and ranking of antioxidant potential – whether FRAP was comparable to the quenching of 2,2-diphenyl-1-picrylhydrazyl (DPPH) in a simple ORAC protocol. Finally, we wished to assess whether organic growing systems had an impact on antioxidant potential in these three crops. The antioxidant potential reported in this study was based on the supermarket salad produce available in the United Kingdom to the consumer on the given date.

## Materials and Methods

### Chemical reagents

Chemicals used were of analytical grade: Iron (II) sulfate heptahydrate and iron (III) chloride hexahydrate were obtained from Acros Organics (part of Fisher Scientific U.K. Ltd, Loughborough, U.K.), TPTZ (2,4,6-tripyridyl-s-triazine) was purchased from Fluka (part of Sigma Aldrich Co. LLC, Gillingham, U.K.), Trolox (6-hydroxy-2,5,7,8-tetramethyl-chroman-2-carboxylic acid) from Aldrich Chemistry, DPPH from Sigma Life Science (part of Sigma Aldrich Co. LLC), sodium acetate and trihydrate and methanol were purchased from Sigma Aldrich Co. LLC and ethanol from Fisher Scientific U.K. Ltd. QIAshredders, for obtaining sap from the salad samples, were purchased from Qiagen Ltd (Manchester, U.K.).

### Variation in salad crops

Four bags of washed and ready watercress, rocket and spinach were purchased from two high-street U.K. supermarkets near the laboratory on 17 February 2009, with an expiration date of either 20 or 21 February. The contents of each bag confirmed that they were from more than one country of origin, and were grown either conventionally or organically certified. The entire contents of each bag were ground under liquid nitrogen and stored at −80°C. Eight replicates of each salad sample were used in each assay. This was to get an “on the shelf” nutritional value for nutrition available, in terms of antioxidants, to the consumer when purchased.

The bags were indiscriminately selected from the products on the shelf in the supermarket during the same time period making it representative of consumer availability. Limitations such as environmental variables are acknowledged but the purpose of this study was to assess what was available to the consumer at the time of purchase.

### Sap extraction

Plant material was ground in liquid nitrogen using a mortar and pestle. As it is important to ensure that the material does not defrost, the material was kept on dry ice. QIAshredders were labeled and weighed before being filled with ground material from each of the lines and the weight of the ground material was recorded. The material was then spun at 13,000 rpm before the extracted sap was pipetted into a fresh eppendorf and the final weight recorded.

### Ferric reducing antioxidant power

FRAP assay is based on the rapid reduction in ferric-tripyridyltriazine (Fe^III^-TPTZ) by antioxidants present in the samples forming ferrous-tripyridyltriazine (Fe^II^-TPTZ), a blue-colored product (Benzie and Strain 1996). A standard curve was created by adding the FRAP reagent to a range of Fe^2+^ solutions of known concentrations which allows the Fe^2+^ concentration of the samples to be calculated thereby determining “antioxidant capacity.” The FRAP method was based on that of Benzie and Strain (1996). Standard solutions of iron (II) sulfate heptahydrate ranging in concentration from 0.25 to 8 mmol dm^−3^ were made. Solutions to make up the FRAP reagent were prepared: 300 mmol/L acetate buffer, 10 mmol/L TPTZ/HCL solution, and 20 mmol/L ferric chloride. A 96-well plate was used for the assay; sap samples were diluted directly into wells. The FRAP reagent was produced using the acetate buffer, TPTZ/HCL and ferric chloride hexahydrate solution and then added to each well. Absorbance was measured directly at 620 nm.

### Oxygen radical absorbance capacity

The procedure was devised by Zullo and Ciafardini (2008) and was followed with reference to that by Kim et al. (2005) and Miliauskas et al. (2004). Quenching of the radical by addition of antioxidants causes a decrease in absorbance at 517 nm due to decolorization as DPPH is reduced to DPPH-H (Friaa and Brault 2006). The assay therefore measures the change in absorbance when DPPH is mixed with the sample. A 0.3 mmol/L solution of the free radical was prepared and then kept in the dark. Set known volumes of sap samples were diluted in eppendorfs and then DPPH was added and mixed thoroughly for 30 sec and then incubated for 10 min in the dark at room temperature. After incubation, reaction mixtures were rapidly transferred to cuvettes and absorbencies measured at 517 nm; the spectrophotometer was first blanked with a solution of water and 80% methanol. The percentage of radicals scavenged was calculated by the following equation (Miliauskas et al. 2004):


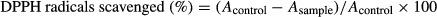



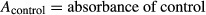



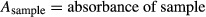


### Statistical analysis

Statistical analysis was carried out using the software Minitab 15 English (Minitab Ltd, Coventry, U.K.). All confidence limits were set at 95%. Analysis was performed using the general linear model (GLM) and significant differences were identified using Tukey's post hoc test.

## Results and Discussion

### Antioxidant analysis of watercress, spinach, and rocket (conventional and organic) using FRAP

The FRAP assay is the only assay that directly measures antioxidants (or reductants) in a sample compared to other assays measuring inhibition of free radicals (Halvorsen et al. 2002). The values expressed from the FRAP assay represent the corresponding concentration of electron-donating antioxidants with the reduction in the ferric iron (Fe^3+^) to the ferrous ion (Fe^2+^) (Halvorsen et al. 2002). FRAP is deemed a suitable assessment for total antioxidants in plants which are consumed by humans because the only compounds with which FRAP does not react with are the thiols. Only a limited amount of glutathione in plants is absorbed by humans with almost no other antioxidant thiols present' in dietary plants (Halvorsen et al. 2002).

**Figure 1:**
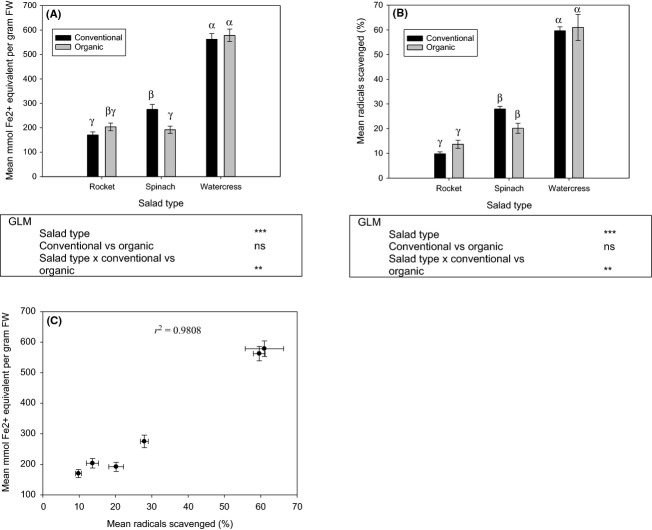
(A) Mean mmol Fe^2+^ equivalent per gram fresh weight in three different salad samples (*F*_2_, _47_ = 231.26, *P* < 0.001) and a comparison between organic and conventional produce (*F*_1_, _47_ = 0.49, *P* > 0.05) (B) mean percentage radicals scavenged in three different samples (*F*_2_, _47_ = 187.2, *P* < 0.05) and a comparison between organic and conventional produce (*F*_1_, _47_ = 0.07, *P* > 0.05). Asterisks identify significant treatment effects from the GLM. **, *P <* 0.01; ***, *P <* 0.001. Bars represent standard error and same symbol denotes no significant difference (Tukey's post hoc test) (C) correlation (*r*^2^ = 0.9808) between the FRAP and ORAC assay with three different salad samples (rocket, spinach, and watercress) and two different production methods (conventional and organic). Bars represent standard error. GLM, general linear model; FRAP, ferric reducing antioxidant power; ORAC, oxygen radical absorbance capacity.

The Fe^III^-TPTZ complex was reduced most strongly to the ferrous (Fe^II^) by conventional watercress 562.4 mmol Fe^2+^ equivalent per gram fresh weight (FW) and organic watercress 578.4 mmol Fe^2+^ equivalent per gram FW (Fig. 1A). Spinach had a lower antioxidant activity (275.0 mmol Fe^2+^ equivalent per gram FW conventional and 191.9 mmol Fe^2+^ equivalent per gram FW organic spinach) compared to watercress, and rocket was lower than spinach, with the exception of the organic rocket (169.9 mmol Fe^2+^ equivalent per gram FW conventional rocket and 203.6 mmol Fe^2+^ equivalent per gram FW organic rocket). It is important to note that the organic spinach had a significantly lower antioxidant activity than conventional spinach while there was no significant difference between organic and conventionally grown watercress and rocket. The different salad samples differed significantly (*F*_2,42_ = 231.26, *P* < 0.001) in their antioxidant activity and a significant interaction (*F*_2,42_ = 5.23, *P* < 0.05) between the crop and cultivation technique was identified.

### Antioxidant analysis of watercress, spinach, and rocket (conventional and organic) using ORAC

The highest percentage of radicals scavenged (Fig. 1B) was observed in the watercress samples (59.59% and 61.00%) followed by spinach (27.95% and 20.18%), and then rocket (9.81% and 13.68%) which reflects the results obtained from FRAP. The salad samples significantly differed in their ability to scavenge radicals (*F*_2,42_ = 187.2, *P* < 0.05) and a significant interaction (*F*_2,42_ = 7.29, *P* < 0.05) between the crop and cultivation technique was observed.

It appears that the different antioxidant assays rank the salad samples in the same order watercress>spinach>rocket. In the FRAP and ORAC assays, organic cultivation had no significant impact on antioxidants, except for spinach. Few studies have been published which compare the nutritional quality provided by conventional and organic produce, although Williams (2002) reported a consumer perception that organic produce results in a higher nutritional quality compared to the conventional produce with superior sensory attributes due to the lower levels of pesticides and synthetic fertilizers used during cultivation. Moreover, conventionally produced vegetables, mainly leafy green vegetables, have a higher nitrate and lower vitamin C content compared to organic produce (Williams 2002). The vitamin C content in spinach and other vegetables is negatively correlated with nitrogen availability with Mozafar (1996) and Magkos et al. (2003) reporting little evidence to support a difference in the concentrations of various micronutrients (vitamins, minerals, and trace elements) between organic and conventional produce. However, there is a trend toward higher ascorbic acid content in organically grown leafy vegetables and potatoes (Magkos et al. 2003). Williams (2002) called for more studies which compare the benefits of conventional and organic produce on human health and the importance of assessing growing conditions on the levels of protective phytochemicals. Tarozzi et al. (2006) showed that organic red oranges had a higher phytochemical content (in terms of phenolics and ascorbic acid), total antioxidant activity, and protective effect against oxidative damage at a cellular level when compared with the conventional red oranges. In contrast to the reported findings of a beneficial impact of organic cultivation on antioxidant activity, this study reveals no impact of organic cultivation on antioxidant potential in watercress and rocket. Moreover, there was a negative impact of organic cultivation on antioxidant potential in spinach.

There is still much debate on the effects of organic and conventional cultivation on photochemical quality and quantity, emphasizing the requirement of research focused on specific crop types and growing conditions (Magkos et al. 2003).

### Correlation between the percentage of radicals scavenged and formation of Fe^II^-TPTZ

There was a clear correlation (*r*^2^ = 0.9808) between the percentage of radicals scavenged in the simplified ORAC assay with the formation of Fe^II^-TPTZ in the FRAP assay (Fig. 1C). As the percentage of radicals scavenged increased so did the formation of Fe^II^-TPTZ in the sample, which supports the idea that both methods reflect total antioxidant activity (Ou et al. 2002). It was clear that watercress ranked highest for antioxidant content followed by spinach and finally rocket with both FRAP and ORAC supporting these findings. This is in contrast to Ou et al. (2002) who reported that FRAP and ORAC values did not correlate well, for antioxidant activity of 927 freeze-dried vegetable samples. It was reported that the ORAC and FRAP values were not only dependent on species but also the geographical origin and harvest time (Ou et al. 2002). It was concluded that the variability between the methods was due to the chemistry behind the assays in which the ORAC method was chemically more relevant to chain-breaking antioxidant activity (Ou et al. 2002). They also reported drawbacks of FRAP such as inference, reaction kinetics, and quantitation methods (Ou et al. 2002).

However, in contrast to Ou et al. 2002, using our simplified ORAC protocol and FRAP there was indeed a clear correlation (*r*^2^ = 0.9808) between the antioxidant amount for the salad samples tested. As the percentage of free radicals scavenged in the ORAC assay increases so does the formation of the Fe^2+^ complex. Martinez-Sanchez et al. (2008) reported a high correlation between ABTS (2,2‘-azino-bis(3-ethylbenzothiazoline-6-sulphonic acid)), FRAP, and DPPH values. With regard to the ABTS and FRAP assay, watercress had the highest milligram 100 g^−1^ antioxidant reading and salad rocket the lowest (Martinez-Sanchez et al. 2008). This reinforces our findings that watercress had the highest and rocket the lowest reducing power (i.e., Fe^3+^-TPTZ to Fe^2+^-TPTZ) and radical scavenging ability.

Further work to underpin the phytochemical composition of watercress, spinach, and rocket will allow the identification of phytochemicals which result in high antioxidant activity.

## Conclusions

We have shown that the antioxidant status of different supermarket-purchased leafy salad crops varies across species and cultivation techniques. Watercress had the highest antioxidant status, when considering the water-soluble antioxidants followed by spinach and wild rocket. We have shown that FRAP and a simplified ORAC assay provide comparable results. In conclusion, this study highlights significant variation in antioxidants between rocket, spinach, and watercress, a strong correlation between two antioxidant assays, FRAP and ORAC, with the impact of organic cultivation varying between species. In particular, we report that supermarket-purchased, organically cultivated spinach may have a lower antioxidant potential, but no impact of organic cultivation on antioxidant status was found for watercress and rocket.
